# 

**Published:** 1883-05-20

**Authors:** 


### TABLE OF CONTENTS.

ORIGINAL AND SELECTED ARTICLES.

Kentucky Medical Society, 161. Thirty-fourth Session of the Georgia State Medical Association, 164. Observations on the Management of Enteric Fever, etc., by Jas. C. Wilson, M. D., 167. Observations in the Larynx and Trachea, by E. Fletcher Ingals, A. M., M. D, 171. Gallstones Successfully treated with Olive Oil, 175. Koch's Reply to his Critics, 176. Pneumonia, 178.

ABSTRACTS AND GLEANINGS.

Colic in Children, 179. Treatment of Spermatorrhœa, 180. Poisoning by Carbolic acid; An Insect that Secretes Prussic Acid, 181. Dyspepsia; Typhoid Fever, 182. Electric Light in Surgery; Treatment of Placenta Pvævia, 183. A Needle Five Years in the Body, 184. New Treatment of Dysentery; A Prognostic Sign of Pneumonia, 185.—Quinine in Whooping-Cough; Hypertrophy of Tonsils, 186. Bicarb Soda for Burns; Greasing with Fat Bacon for Scarlatina; Mineral Acids in Summer Diarrhœa, 187. Dr. Cattaneo's Treatment of Hydrocele, 188. Salicylate of Soda in Typhoid Fever; M. Pasteur; Powdered Capsicum, 189.

SCIENTIFIC ITEMS.

Time by Telegraph; Iron Shot; Melting Steel; A Non-Conductor, 190. An Instantaneous Light; Among; It is well known, 191.

PRACTICAL NOTES AND FORMULÆ.

Cardiac Neurasthenia; Medication in Uterine Affections; Dysentery, 192. Remedy for Constipation; Bilious Dysentery, 193. Novel Method of taking Quinine; Acute Bronchitis; Diarrhœa, 194.

EDITORIALS AND MISCELLANEOUS.

Editorial Notices; Medical Association of Alabama; Medical Association of Georgia, 195. Mineral Waters—their Medicinal and Hygienic Advantages, 196. Koch's Bacillus of Tubercle; In Memoriam, 198. Book Notices; Pamphlets Received; Receipted, 199. Special Notices, 200.

